# Chronic stress synergizes with *Listeria monocytogenes* to promote intestinal adenomagenesis via myeloid-derived suppressor cells

**DOI:** 10.3389/fimmu.2025.1653548

**Published:** 2025-09-03

**Authors:** Pingqian Qi, Lili Yin, Ruijia Wei, Siyuan Yang, Ziqing Liu, Ping Huang, Qiwen Yu, Suyi Xiong, Mengmeng Wang, Yanjuan Deng, Jinping Hu, Lv Zhou, Ruishan He, Huan Deng, Ying Xiong

**Affiliations:** ^1^ Department of General Medicine, The Second Affiliated Hospital, Jiangxi Medical College, Nanchang University, Nanchang, Jiangxi, China; ^2^ Department of Pathology, Affiliated Rehabilitation Hospital, Jiangxi Medical College, Nanchang University, Nanchang, China; ^3^ Tumor Immunology Institute, Nanchang University, Nanchang, Jiangxi, China; ^4^ Department of Radiotherapy, First Hospital of Tsinghua University, Beijing, China; ^5^ The Ministry of Education (MOE) Basic Research and Innovation Center for the Targeted Therapeutics of Solid Tumors, Affiliated Rehabilitation Hospital, Jiangxi Medical College, Nanchang University, Nanchang, China

**Keywords:** *Listeria monocytogenes*, MDSCs, adenoma, intestinal barrier, chronic stress

## Abstract

**Background:**

Chronic stress and gut dysbiosis are established risk factors for colorectal adenocarcinoma, yet their synergistic effects on the development of intestinal precancerous lesions remain poorly understood.

**Methods:**

This study investigates the molecular mechanisms through which chronic stress interacts with opportunistic pathogen *Listeria monocytogenes* to drive intestinal tumorigenesis in *Apc* ^Min/+^ mice, with particular focus on the involvement of tumor immune microenvironment remodeling.

**Results:**

The combination of *L. monocytogenes* infection and chronic stress, rather than bacterial infection alone, significantly increased colonic adenoma burden and epithelial dysplasia, suggesting that chronic stress establishes a permissive microenvironment for opportunistic pathogens to exert pro-tumorigenic effects. Mechanistically, chronic stress downregulated intestinal epithelial *Muc-2* expression and reduced microbial diversity, thereby compromising mucus/microbial barrier integrity and enhancing *L. monocytogenes* colonization. Under dual stress-pathogen exposure, we observed the expansion of myeloid-derived suppressor cells (MDSCs) in spleen and the upregulation of IL-6 in colonic mucosa, which facilitated MDSCs recruitment to tumor sites. Infiltrating MDSCs driven CD8^+^ T cell depletion through cAMP/PKA/CREB signaling, leading to the establishment of immunosuppressive microenvironment.

**Conclusion:**

Our results propose that chronic stress-induced gut barrier disruption may serve as a prerequisite for opportunistic pathogens to accelerate the development of precancerous lesions. Their synergistic effects reshape systemic/local immune responses, creating a microenvironment conducive to malignant transformation and tumor cell survival. These preliminary findings highlight potential clinical applications of psychological interventions and immune modulation strategies in preventing intestinal carcinogenesis.

## Introduction

Colorectal adenocarcinoma (CRC) ranks as the third most prevalent malignancy and represents the second leading cause of cancer-related mortality globally (1–3). The colorectal adenoma, a benign glandular tumor, is a precursor lesion of CRC (2). As the only currently effective treatment for colorectal adenoma, colonoscopic resection has been proven to significantly reduce the incidence of CRC. However, high recurrence rates of adenoma remain the biggest obstacle to progress in complete healing (4–6).

Insufficient understanding of pathogenesis represents an important factor limiting our efforts to develop therapeutic interventions against adenoma. Considering the pathological continuum from chronic inflammation to tumor, intestinal dysbiosis may stand at the crossroad and contribute to the colonization of opportunistic pathogen (7). Opportunistic pathogens such as *Fusobacterium nucleatum* and *Streptococcus gallolyticus* can promote the development of intestinal adenoma through various mechanisms, including disrupting intestinal barrier function, inducing chronic inflammatory responses, and modulating the immune microenvironment (7–9).

*Listeria monocytogenes* (*L. monocytogenes*), a common opportunistic pathogen, is widely distributed in natural environments and food products (10). Clinical studies have revealed that *L. monocytogenes* is significantly enriched in tumor tissues of CRC patients compared to adjacent normal tissues (11), suggesting a potential spatiotemporal association between *L. monocytogenes* and CRC. However, there have been no reports addressing whether *L. monocytogenes* participates in driving colorectal precancerous lesions, considering the pathological continuum from adenoma to adenocarcinoma. Notably, as *L. monocytogenes* is more prone to cause systemic infections in immunocompromised individuals, it has been suggested to preferentially colonize tissues under the immunosuppressive microenvironment (12, 13). Moreover, unlike common CRC-associated pathogens such as *Fusobacterium nucleatum*, *L. monocytogenes* preferentially colonizes in immunosuppressive microenvironments. Surface proteins of *L. monocytogenes* have been shown to disrupt intestinal epithelial tight junctions (TJs) and facilitate trans-epithelial transport within 24 hours (14). Critically, *L. monocytogenes* can induce IL-6 production via the TLR2/NF-κB and MAPK pathways, thereby recruiting myeloid-derived suppressor cells (MDSCs) (15). However, the specific pathophysiological contexts through which *L. monocytogenes* contributes to adenomagenesis require systematic investigation.

Recent studies have increasingly recognized the fact that mental disorders have etiologic impact on the pathogenesis of chronic diseases and tumors (16). Both direct such as neuroendocrine pathways and indirect mechanisms, including gut microbiota dysbiosis, impaired intestinal barrier function, and tumor immune microenvironment dysregulation, are involved in this process (17, 18). However, direct experimental evidence linking these mechanisms to preneoplastic lesions remains conspicuously absent (19, 20). The immune system and mental disorders are intricately linked. Recent research suggests that MDSCs, a heterogeneous population of bone marrow-derived cells with significant immunosuppressive functions, may also play a central role in the immune dysregulation associated with mental disorders, such as major depression (21). Accumulating evidence has documented that MDSCs exhibit marked infiltration within the tumor microenvironment (TME) of colonic adenoma, where they potently suppress CD4^+^/CD8^+^ T cells directly or through Tregs, thereby facilitating immune evasion of neoplastic cells (22, 23). Moreover, preclinical studies across multiple tumor models have corroborated that psychological stress potentiates both recruitment and functional activation of MDSCs via sympathetic nervous pathway (24–26). However, the potential involvement of opportunistic pathogens in remodeling the immune microenvironment to impact intestinal adenomagenesis under chronic stress conditions remains a mechanistically unresolved paradigm.

In this study, we established a dual-perturbation model integrating chronic stress and *L. monocytogenes* infection in *Apc* ^Min/+^ mice to mechanistically delineate how the neuro-microbe-immune triad orchestrates molecular pathways governing premalignant lesion initiation and development within the colonic microenvironment. The findings demonstrate that chronic stress compromises intestinal barrier integrity and enhances mucosal colonization capacity of *L. monocytogenes*. These dual perturbations synergistically drive splenic expansion and colonic recruitment of MDSCs. Infiltrating MDSCs impair CD8^+^ T cell functions via the cAMP/PKA/CREB signaling axis, thereby enhancing the immunosuppressive capacity of TME to facilitate adenomagenesis. This study elucidates the mechanistic interplay between neuropsychological stress and opportunistic pathogens in orchestrating tumor-permissive microenvironments, providing novel molecular basis and attractive targets for the diagnosis and treatment of intestinal adenoma.

## Materials and methods

### Mice

C57BL/6 background *Apc* ^Min/+^ mice have been described previously (27). Mice were observed carefully by laboratory staff and veterinarian personnel for health and activity. Mice were monitored to ensure that food and fluid intake meets their nutritional needs. Body weights were recorded at minimum weekly, and more often for animals requiring greater attention. Mice were maintained on wood chip bedding, and given *ad libitum* access to water and standard mouse chow, with 12-hour light/dark phase cycles. The colonies were specific pathogen free (SPF) and tested quarterly for known pathogens. Mice in the barrier facilities are housed in cages with microisolator tops on ventilated or static racks. All caging materials and bedding are autoclaved. Food is irradiated and water is either RO, autoclaved or acidified, depending on the barrier. All manipulations are performed in laminar flow hoods. Once animals are removed from a barrier, they are not returned. All personnel wear shoe covers, gloves, hair bonnets and gowns. All mouse studies were approved by the Nanchang University Institutional Animal Care and Use Committee (No. SYXK2021-0004).

### Chronic stress model

Adenoma formation in *Apc* ^Min/+^ mice can be detected as early as 5 weeks of age (28). In this study, 7-week-old *Apc* ^Min/+^ mice were randomly assigned to a control group (Ctrl) and a chronic stress group (CS). Mice in the CS group underwent 4-hour daily restraint stress for 10 consecutive days, while the control group was maintained under standard environmental exposure conditions (29). Body weight was monitored daily throughout the experimental period. On day 11, anxiety-like behaviors induced by chronic stress were evaluated using the open field test (OFT) and elevated plus maze (EPM), which exploit rodents’ innate aversion to exposed spaces, where unprotected or open areas function as anxiogenic stimuli. In these paradigms, anxiety-like behavior and exploratory activity are quantified by measuring time spent in each zone and total distance traveled during the test sessions (30, 31).

OFT and EPM were utilized to assess chronic stress-induced anxiety-like behaviors in mice. Behavioral parameters in OFT, including total distance traveled, locomotor trajectories, and duration in the central zone, were automatically tracked and analyzed using Any-Maze software. The EPM apparatus consisted of two open arms (27 × 5 cm), two closed arms (27 × 5 cm), and a central platform (5 × 5 cm). During testing, mice were gently placed on the central platform facing an open arm and allowed to freely explore the maze for 10 minutes. Behavioral metrics for EPM, such as total distance traveled, movement trajectories, and time spent in the open arms, were subsequently quantified. Behavioral metrics in EPM, including movement trajectories, their entries into the open arms, and time spent in the open arms, were quantified.

### *L. monocytogenes* infection

*L. monocytogenes* (strain ATCC 19115), kindly provided by Dr. Hengyi Xu, was revived overnight on PALCAM agar plates (HopeBiol, Qingdao, China) at 37°C. A single colony was inoculated into Tryptic Soy Broth (TSB) supplemented with 0.6% yeast extract (HopeBiol, Qingdao, China) and incubated under shaking (200 rpm) at 37°C overnight. The bacterial culture was centrifuged (8,000 ×g, 10 min, 4°C) the following day, and the pellet was resuspended in sterile phosphate-buffered saline (PBS) to adjust the optical density to OD_600_ = 1.0.

Following successful establishment of the chronic stress model, mice were stratified into 4 groups based on *L. monocytogenes* infection status: Ctrl (control), LM (*L. monocytogenes* infection), CS (chronic stress), and CS+LM (chronic stress and *L. monocytogenes* infection). On day 12 of the experiment, mice in the LM and CS+LM groups were orally gavaged with 200 μL of *L. monocytogenes* suspension (OD_600_ = 1.0, ~10^8 CFU), while Ctrl and CS groups received an equal volume of sterile PBS. After 24 hours of bacterial colonization, all mice were euthanized by cervical dislocation. Spleen, colon, and fecal samples were collected for analyses.

### Histopathological analysis

Colon mucosal and adenoma tissues were harvested and fixed overnight in 4% paraformaldehyde solution. The tissues were subsequently dehydrated through graded alcohol solutions. Following dehydration, the tissues were embedded in paraffin wax. Sections of 5 μm thickness were prepared using a microtome and subjected to hematoxylin and eosin (HE) staining (Solarbio, Beijing, China). Epithelial cell morphology, glandular architecture, and crypt structures were observed and analyzed under an optical microscope by two independent pathologists (Dr. Mei Li and Qiong Feng).

### Flow cytometry analysis

Single-cell suspensions from mouse spleen, colon, and adenoma tissues were prepared to assess the proportions of different immune cell populations using flow cytometry. Spleen tissues were mechanically homogenized, subjected to red blood cell lysis, and filtered through a 70 μm nylon mesh to obtain single-cell suspensions. Colon tissues were minced and digested in RPMI 1640 medium (Thermo Fisher, Massachusetts, USA) containing collagenase I (Beyotime, Shanghai, China) and DNase I (Sangon Biotech, Shanghai, China) at 37°C in a shaking incubator for 30 minutes, followed by filtration through a 70 μm nylon mesh. To reduce nonspecific binding and background fluorescence, Fc receptor blocking reagent (BD Biosciences, New Jersey, USA) was added to the single-cell suspensions (≤1×10^6^ cells). Antibodies targeting specific immune markers (**Supplementary Table S1**) were then incubated with the cells based on their immunophenotypes: MDSCs: Identified as CD45^+^CD11b^+^GR1^+^. CD8^+^ T cells: Identified as CD3^+^CD8^+^CD4^−^. Samples were analyzed using a Beckman CytoFLEX flow cytometer (CA, USA), and data were processed with FlowJo 10.6.2 software.

### ELISA detection

Fresh mouse colon tissues were homogenized in RIPA lysis buffer (Sangon Biotech, Shanghai, China) containing protease inhibitors (Ncmbio, Suzhou, China) using a cryogenic grinder (Jingxin, Shanghai, China). After low-temperature centrifugation, supernatants were collected, and protein concentrations were quantified using a BCA assay (Beyotime, Shanghai, China). Levels of interleukin-6 (IL-6; Beyotime, Shanghai, China) and cyclic adenosine monophosphate (cAMP; Cayman Chem, Michigan, USA) in colon tissues were measured according to the manufacturers’ protocols.

### RNA-seq and analysis

Total RNA was extracted from mouse colon tissues using the Trizol method. RNA quality and concentration were assessed via NanoDrop spectrophotometry (Thermo Fisher, Massachusetts, USA). Eukaryotic transcriptome libraries were constructed using the Hieff NGS^®^ Ultima Dual-mode mRNA Library Prep Kit (12309ES, Yeasen, Shanghai, China). Library quality was verified with a DNA 1000 Assay Kit (Agilent Technologies, 5067-1504) or High Sensitivity DNA Assay Kit (Agilent Technologies, 5067-4626). Sequencing data were analyzed on the Omicsmart platform (www.omicsmart.com). Sequencing services were provided by Gene Denovo Co., Ltd (Guangzhou, China).

Principal Component Analysis (PCA) evaluated intra-group reproducibility and inter-group differences. Differentially expressed genes (DEGs) were identified using volcano plots with thresholds of |log2FoldChange| > 2 and FDR < 0.05. Venn diagrams visualized shared and unique DEGs across groups. KEGG pathway analysis was performed to annotate biological functions of DEGs.

### Western blotting analysis

Total protein was extracted from colon tissues, and concentrations were determined using a BCA Protein Assay Kit (Beyotime, Shanghai, China). Proteins were separated by SDS-PAGE and transferred to PVDF membranes. Membranes were blocked with 5% skimmed milk, incubated with primary antibodies, followed by horseradish peroxidase (HRP)-conjugated secondary antibodies. Signals were detected using a ChemiDoc TM XRS+ imaging system (Bio-Rad, USA) with chemiluminescent substrate (Uelandy, Suzhou, China). Band intensities were quantified via ImageJ software and normalized to β-tubulin. Antibodies included: anti-PKA (1:3000), anti-CREB (1:3000), and anti-β-tubulin (1:7000) (Immunoway, Texas, USA).

### Quantitative RT-PCR

Total RNA was extracted from colon tissues using Trizol reagent. cDNA was synthesized using Hifair^®^ III 1st Strand cDNA Synthesis SuperMix for qPCR (Yeasen, Shanghai, China). Gene expression levels of Mucin-2, Occludin, Zonula Occludens-1, and Claudin3 were amplified using Hieff UNICON^®^ Universal Blue qPCR SYBR Green Master Mix (Yeasen, Shanghai, China). mRNA levels were normalized to GCRSPH (primers synthesized by Sangon Biotech, Shanghai, China; see **Supplementary Table S2**).

### 16S rDNA sequencing

Total bacterial DNA from mouse fecal samples was extracted using the HiPurA Stool DNA Purification Kit (Magen, Guangzhou, China). DNA quality and concentration were assessed via NanoDrop spectrophotometry. The V3-V4 hypervariable regions of bacterial 16S rRNA genes were amplified with primers 341F (CCTACGGGNGGCWGCAG) and 806R (GGACTACHVGGGTATCTAAT). Valid tags were clustered into operational taxonomic units (OTUs) at 97% similarity using the UPARSE pipeline (v9.2.64). Sequencing was performed on an Illumina HiSeq 2500 platform (PE250; Gene Denovo Co., Ltd, Guangzhou, China). Data analysis was conducted on the Omicsmart platform. Alpha diversity indices (ACE, Sob, Shannon) were calculated based on OTU richness. Beta diversity was visualized via PCoA and NMDS using unweighted UniFrac distances. Taxonomic composition (phylum and genus levels) was displayed as stacked bar plots. Welch’s *t*-test identified species with significant abundance differences between groups.

### Intestinal permeability analysis

After fasting for 6 hours, mice were orally gavaged with 200 μL of 600 mg/mL fluorescein isothiocyanate-dextran (FITC-dextran; Sigma-Aldrich, Missouri, USA). Blood was collected 4 hours post-administration. Serum FITC-dextran concentrations were measured after centrifugation.

### Statistical analysis

Data were analyzed and visualized using GraphPad Prism 9.5. Results are expressed as mean ± SEM. Two-group comparisons: Unpaired Student’s *t*-test (normally distributed data with equal variance) or Mann-Whitney *U* test (nonparametric data). Multi-group comparisons: Kruskal-Wallis *H* test (nonparametric data). Significance was defined as P < 0.05.

## Results

### CS/LM synergistically promotes colonic adenomagenesis

**Figure 1:**
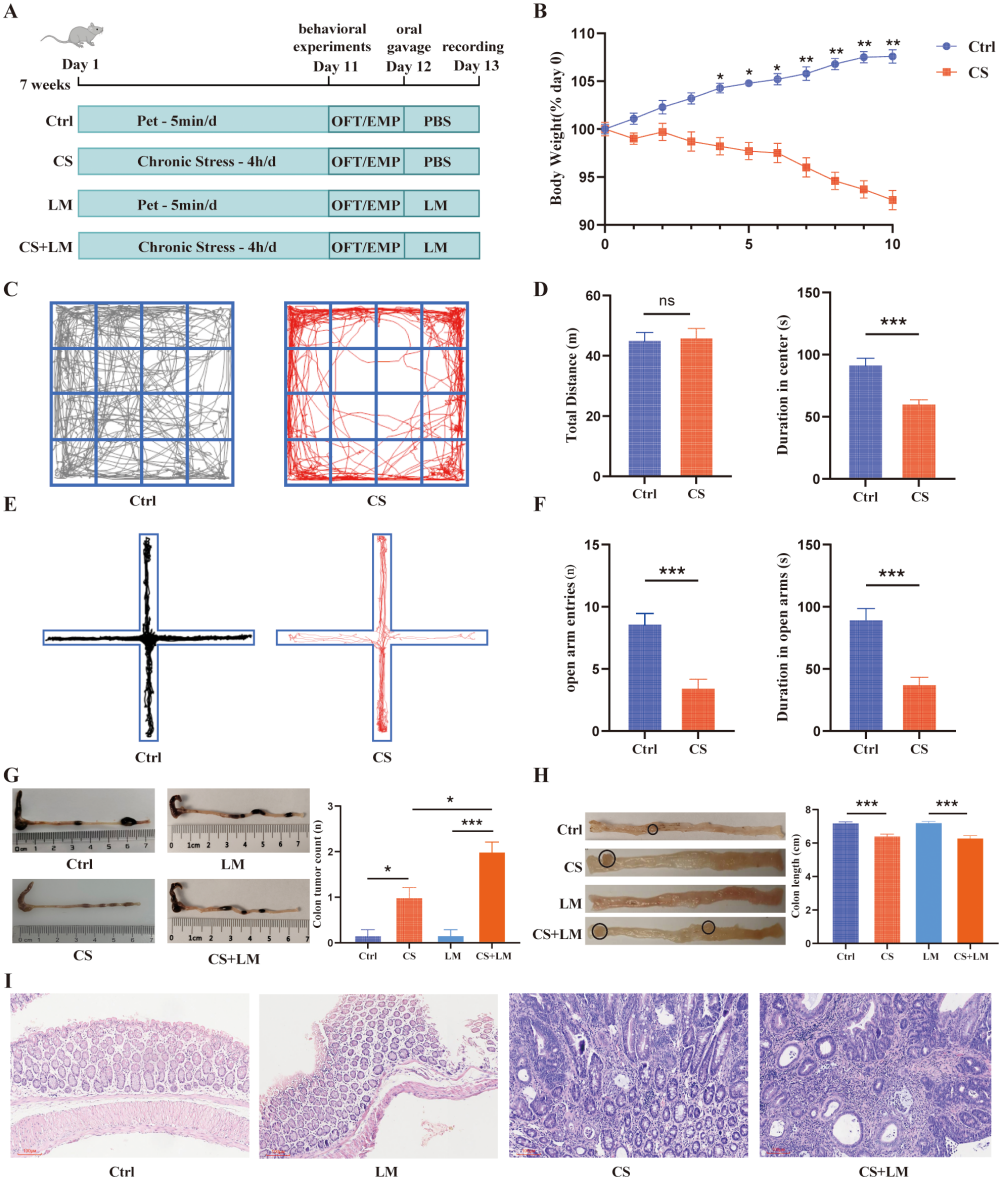
*L. monocytogenes* cooperates with chronic stress to promote intestinal adenoma formation. **(A)** Schematic diagram of the chronic stress and LM infection model. **(B)** Chronic stress caused a significant reduction in mouse body weight (n=6). **(C)** Representative locomotion traces from the open field test. **(D)** Spontaneous locomotor activity assessed by the open field test, comparing the total distance traveled (left) and time spent in the central zone (right) (n=12). **(E)** Locomotion traces from the elevated plus maze test. The horizontal arms are open, while the vertical arms are closed. **(F)** Spontaneous locomotor activity assessed by the elevated plus maze test, comparing the entries into the open arms (left) and time spent in the open arms (right) (n=12). **(G)** Colon length obtained from mice in the CS and CS+LM groups was significantly shorter than that in the Ctrl and LM groups (n=6). **(H)** Chronic stress synergized with *L. monocytogenes* to increase the number of colonic adenomas (n=6). **(I)** Chronic stress alone or in combination with *L. monocytogenes* induced intestinal epithelial atypia (original magnification ×100). n.s., not significant; *P < 0.05; **P < 0.01; ***P < 0.001. n indicates biological replicates.

Although prior studies have demonstrated the individual roles of chronic stress and gut microbiota in intestinal tumorigenesis (32–34), their synergistic effects remain unclear. We established a chronic stress model in *Apc* ^Min/+^ mice and validated its success through behavioral tests, including OFT and EPM (**Figure 1A**). Results showed that CS-treated mice exhibited significant weight loss, reduced time spent in the central zone of the OFT, and diminished exploration in the open arms of the EPM, indicating pronounced chronic stress-like behaviors (**Figures 1B–F**).

Upon *L. monocytogenes* infection in CS-exposed mice (**Figure 1A**), we observed that both CS and CS+LM groups displayed markedly shortened colon lengths and increased colonic adenoma counts compared to Ctrl and LM-only groups (**Figures 1G, H**). Notably, while CS+LM group developed more adenomas than CS group, polyp lesions in both groups were histologically classified as high-grade tubular adenomas (**Figure 1I**). These findings suggest that *L. monocytogenes* exacerbates colonic adenoma burden in *Apc* ^Min/+^ mice only under chronic stress conditions, revealing a synergistic tumor-promoting effect between CS and *L. monocytogenes*.

### MDSCs expansion and recruitment

**Figure 2:**
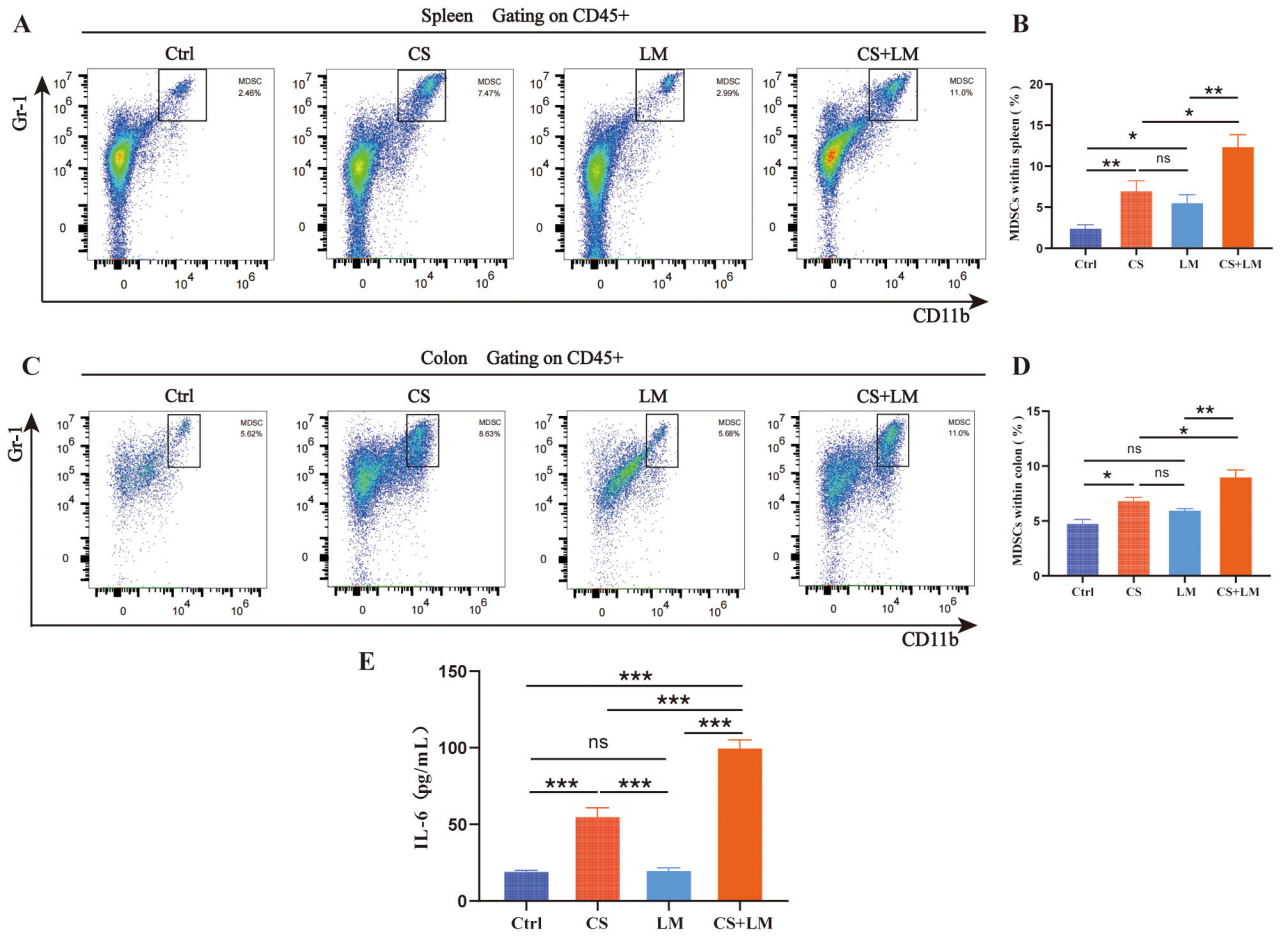
Expansion and recruitment of MDSCs. **(A, B)** Chronic stress, *L. monocytogenes*, or their combination promoted an increase in the proportion of MDSCs within the spleen (n=6). **(C, D)** Chronic stress alone or in combination with *L. monocytogenes*, but not *L. monocytogenes* alone, promoted the infiltration of MDSCs (n=6). **(E)** Chronic stress alone or in combination with *L. monocytogenes* promoted an increase in IL-6 level within the intestinal mucosa (n=6). n.s., not significant; *P < 0.05; **P < 0.01; ***P < 0.001. n indicates biological replicates.

A higher proportion of MDSCs has been detected in tumor tissues of patients with intestinal adenomas and adenocarcinomas (35, 36). Consistent with previous findings (37), flow cytometry revealed that the proportions of CD11b^+^Gr1^+^ MDSCs in both the spleen and colon of CS-treated mice were significantly elevated compared to the Ctrl group (**Figures 2A, B**). Notably, CS/LM co-exposure further increased the proportion of MDSCs within splenic and colonic immune cell populations (**Figures 2A, B**). However, *L. monocytogenes* alone only increased MDSCs in the spleen, not the colon, suggesting that chronic stress is a prerequisite for *L. monocytogenes* to modulate the colonic mucosal microenvironment (**Figures 2C, D**).

IL-6, a key regulator of MDSCs recruitment and activation, exhibits a positive correlation with immunosuppressive cell infiltration in the tumor microenvironment (38, 39). To investigate the molecular mechanism underlying MDSCs recruitment to the colon, ELISA analysis confirmed that CS alone, but not LM, significantly upregulated colonic mucosal IL-6 levels. This aligns with the observation that *L. monocytogenes*-induced MDSCs accumulation in the colon only in the setting of chronic stress. The highest IL-6 levels were detected in the CS+LM group, further supporting the synergistic effect of CS and *L. monocytogenes* in promoting MDSCs expansion and infiltration into the colon (**Figure 2E**).

### cAMP/PKA/CREB pathway enhances the function of MDSCs

**Figure 3:**
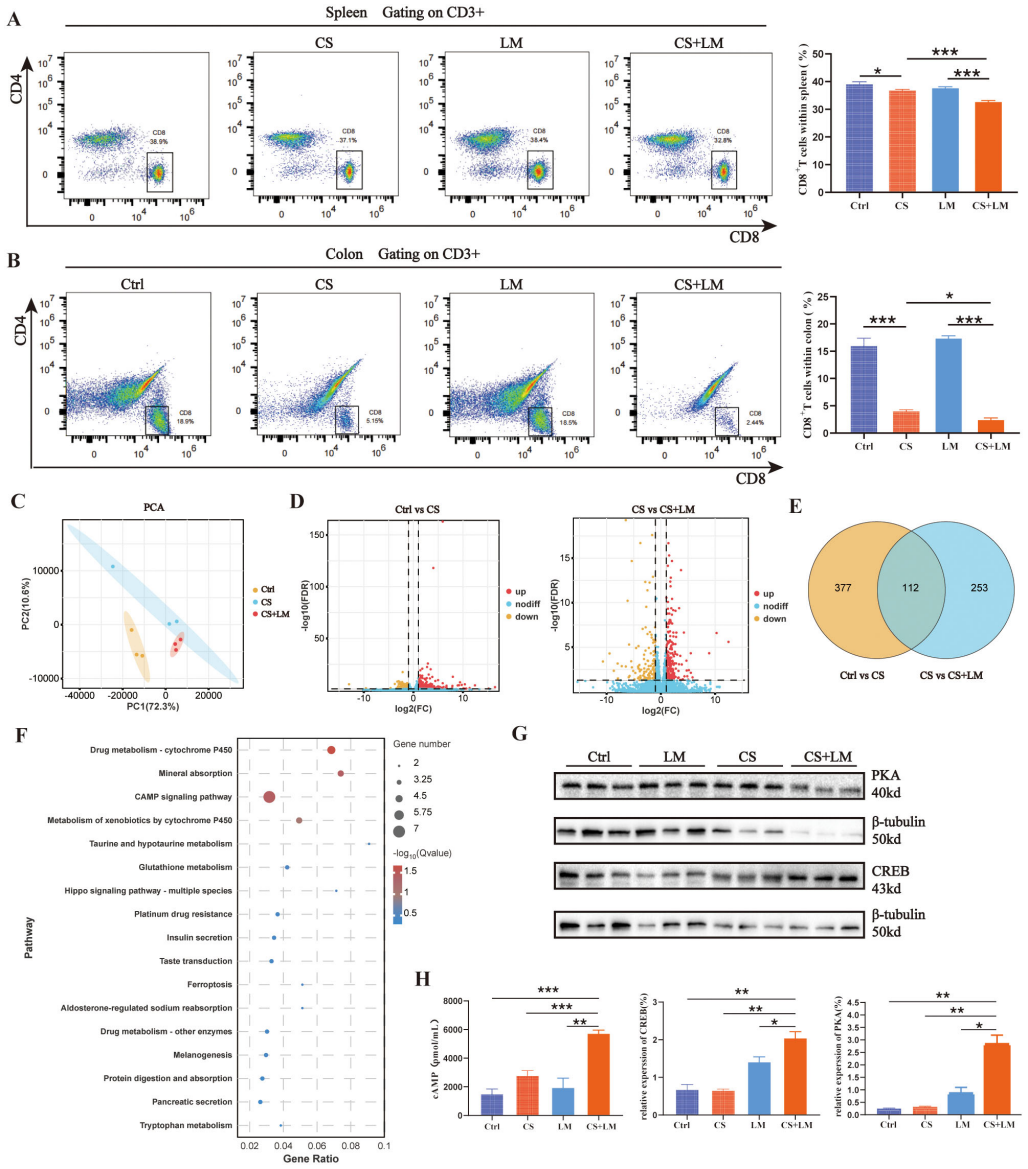
The cAMP/PKA/CREB pathway regulates the immunosuppressive function of MDSCs. **(A)** Chronic stress and *L. monocytogenes* reduce the proportion of CD8^+^ T cells in the spleen (n=6). **(B)** Chronic stress, but not *L. monocytogenes*, decreases the proportion of CD8^+^ T cells in colonic mucosa across groups (n=6). **(C)** PCA results show significant differences between Ctrl and the other two groups, while CS and CS+LM exhibit similar sample composition (n=3). **(D)** Volcano plots of differentially expressed genes (DEGs) between groups. Left: Ctrl vs CS; Right: CS vs CS+LM (n=3). **(E)** Venn diagram displays 112 common DEGs between the two comparisons (Ctrl vs CS and CS vs CS+LM). **(F)** Bubble plot of KEGG pathway enrichment analysis for significant DEGs. **(G)** Western blot analysis of PKA and CREB proteins in colonic tissues (n=3). **(H)** Chronic stress and *L. monocytogenes* induce elevated expression of cAMP, PKA, and CREB in colonic mucosa (n=3 or 4). n.s., not significant; *P < 0.05; **P < 0.01; ***P < 0.001. n indicates biological replicates.

The core function of MDSCs lies in their potent immunosuppressive activity, particularly in promoting regulatory T cells (Tregs) and suppressing effector T cells (23, 40). To investigate changes in MDSCs roles under CS and *L. monocytogenes* infection conditions, we examined CD8^+^ T cell populations in the colon mucosa. Results showed that CS alone, but not LM alone, reduced the proportion of CD8^+^ T cells in both the spleen and colon mucosa, with a more pronounced reduction in the CS+LM group (**Figures 3A, B**). These findings suggest that *L. monocytogenes* infection under chronic stress suppresses CD8^+^ T cell proliferation and infiltration.

We further explored the correlation between MDSCs and T cell behaviors in lesion tissues using transcriptomics. Principal component analysis (PCA) revealed closer clustering between CS and CS+LM groups, indicating similar sample compositions, while both groups diverged significantly from the Ctrl group (**Figure 3C**). Volcano plot analysis identified 489 DEGs in the CS group versus Ctrl, with 365 additional DEGs induced by CS+LM compared to CS alone (**Figure 3D**). A Venn diagram highlighted 112 overlapping DEGs between the two comparison groups (**Figure 3E**). KEGG pathway enrichment analysis showed these DEGs were primarily associated with tumor metabolism, inflammation, and immune-related pathways, including the cyclic adenosine monophosphate (cAMP) signaling pathway, ferroptosis, glutathione metabolism, and mineral absorption. Notably, the cAMP pathway exhibited the highest number of DEGs (**Figure 3F**).

The cAMP signaling pathway is implicated in regulating the immunosuppressive effects of MDSCs within tumors (41, 42). To investigate whether L. monocytogenes activates this pathway to remodel the TME via MDSCs in precancerous lesions under chronic stress, we measured key components of the cAMP pathway, including cAMP, protein kinase A (PKA), and cAMP-response element binding protein (CREB), in the colon mucosa using ELISA and Western blotting. Results demonstrated significantly elevated expression of cAMP, PKA, and CREB in the CS+LM group compared to other groups (**Figures 3G, H**). These data suggest that LM infection under chronic stress activates the cAMP/PKA/CREB pathway, thereby enhancing MDSCs immunosuppressive function, inducing CD8^+^ T cell depletion, and accelerating adenoma progression.

### Chronic stress and *L. monocytogenes* compromise intestinal barrier integrity

**Figure 4:**
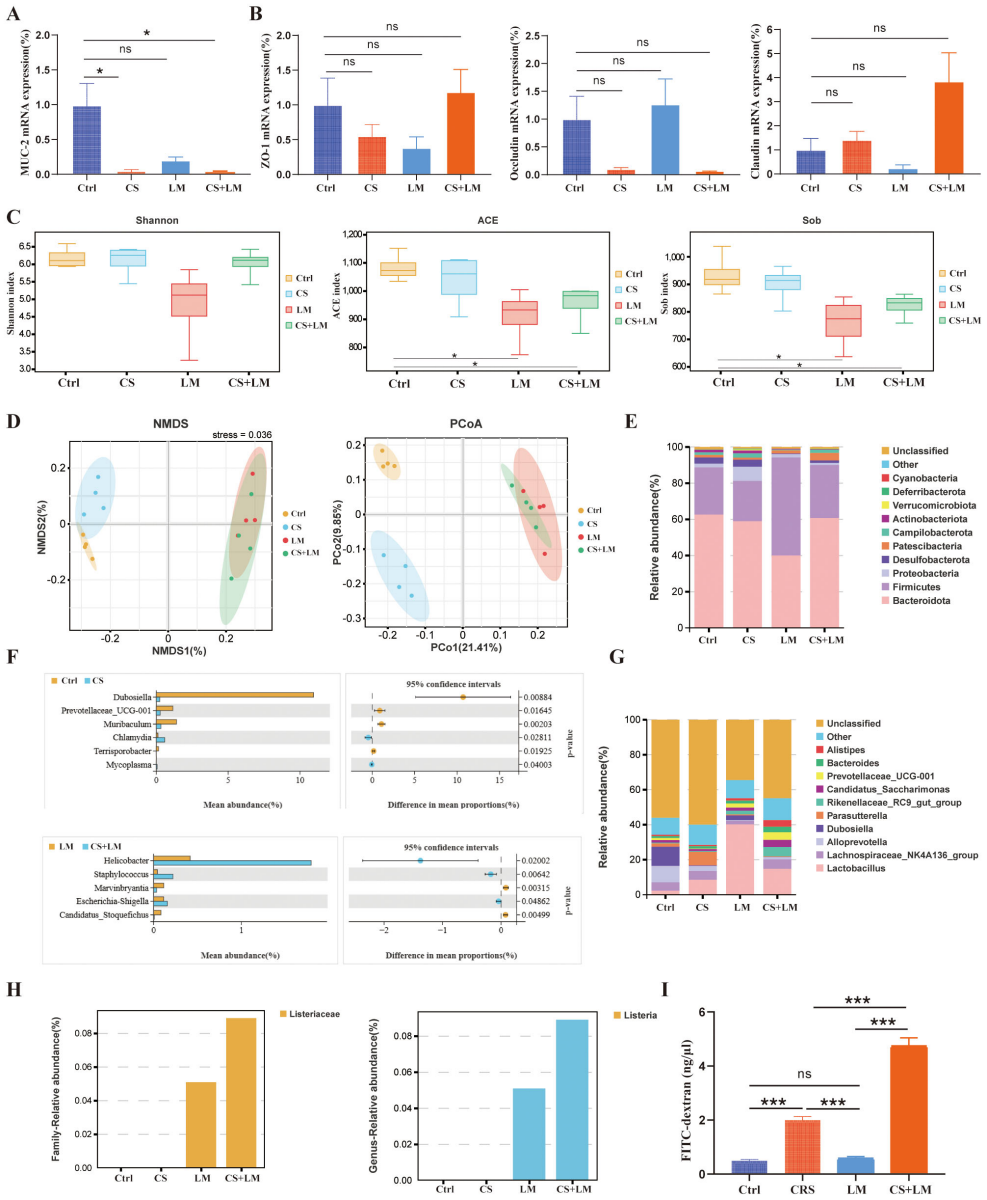
Chronic stress and *L. monocytogenes* compromise the intestinal barrier. **(A, B)** Effects of chronic stress and *L. monocytogenes* on the expression of MUC-2, Occludin, ZO-1, and Claudin3 in colonic tissues (n=4). **(C)** Alpha diversity indices (ACE, Sob, Shannon) assessing community diversity (n=4). **(D)** Differences in fecal microbiome composition among groups evaluated by NMDS (non-metric multidimensional scaling) and PCoA (principal coordinate analysis) (n=4). **(E)** Stacked bar plot showing the cumulative relative abundance of the top 10 bacterial phyla across groups (n=4). **(F)** Differential abundance analysis of bacterial genera between comparison groups (n=4). **(G)** Stacked bar plot showing the cumulative relative abundance of the top 10 bacterial genera (n=4). **(H)** Relative abundance of *Lactobacillus* at the family (left) and genus (right) levels (n=4). **(I)** Intestinal permeability assessed by FITC-dextran assay (n=4). n.s., not significant; *P < 0.05; **P < 0.01; ***P < 0.001. n indicates biological replicates.

As the primary defense of the digestive system, intestinal barrier dysfunction plays a pivotal role in the pathogenesis of chronic inflammation-related CRC (43). The intestinal barrier is collectively composed of mechanical, immune, chemical, and microbial barriers (43). The up-mentioned results suggest that CS and *L. monocytogenes* infection may exert distinct effects on the intestinal mucosa. To further explore the underlying mechanisms, we first examined the expression of key genes associated with the chemical and mechanical barriers. Mucin-2, a major component of the intestinal mucus layer, contributes to the chemical barrier in the colon (44). RT-qPCR results revealed that the expression levels of *MUC-2* (the gene encoding Mucin-2) in the colon tissues of CS+LM and CS group mice were significantly lower than those in the Ctrl group. However, *L. monocytogenes* infection alone did not exhibit a significant impact on *MUC-2* expression (**Figure 4A**).

TJs between colonic epithelial cells serve as the physical foundation of the colonic mechanical barrier. Among these, Occludin, Zonula Occludens-1 (ZO-1), and Claudin-3 are the most crucial protein structures of TJs (43). RT-qPCR results indicated no significant differences in the expression of TJ-related genes compared to the Ctrl group (**Figure 4B**). These findings suggest that CS and *L. monocytogenes* primarily affect the intestinal chemical barrier, rather than the mechanical barrier.

To investigate the status of the intestinal microbial barrier, we collected fecal samples from each group of mice for 16S rDNA gene sequencing. The results revealed that chronic stress had minimal impact on the α-diversity of the gut microbiota, while *L. monocytogenes* infection led to a significant decrease in the richness component of gut microbiota α-diversity (**Figure 4C**). Analyses of β-diversity, including Non-metric Multidimensional Scaling (NMDS) and Principal Coordinates Analysis (PCoA), demonstrated significant separation among the 4 groups, indicating distinct differences in species composition between the groups (**Figure 4D**).

At the phylum level, CS led to a decrease in the abundance of *Firmicutes*. Following *L. monocytogenes* infection, the abundance of *Actinobacteria* decreased (**Figure 4E**). At the genus level, the abundances of *Gluconobacter*, *Bacteroides*, and *Alistipes* increased under chronic stress, whereas *L. monocytogenes* infection did not significantly alter the abundance of these bacterial groups (**Figure 4G**). To further investigate the impact of chronic stress on the microbiota, we performed Welch’s *t*-test to identify differentially abundant genera (**Figure 4F**). Compared to the Ctrl group, CS mice exhibited significantly increased abundances of *Dubosiella*, *Prevotellaceae UCG-001*, *Muribaculum*, and *Terrisporobacter* in their feces, alongside significantly decreased abundances of *Chlamydia* and *Mycoplasma*.

Compared to the LM group, CS+LM mice showed significantly increased abundances of *Helicobacter*, *Staphylococcus*, and *Escherichia-Shigella*, while abundances of *Marvinbryantia* and *Candidatus Saccharimonas* were significantly decreased. Furthermore, analysis of *L. monocytogenes*-affected bacterial families and genera revealed that although normal mice and chronic stress mice were gavaged with an equivalent number of *L. monocytogenes* suspension, the intestinal colonization load of *L. monocytogenes* was significantly higher under chronic stress conditions (**Figure 4H**).

Additionally, intestinal barrier integrity analysis showed the highest serum concentration of FITC-D4000 in the CS+LM group, indicating the greatest intestinal permeability. The CS group had the next highest level, while the Ctrl and LM groups showed the lowest levels with no significant difference between them (**Figure 4I**). These results indicate that CRS primarily affects the integrity of the epithelial cell barrier. On this basis, *L. monocytogenes* synergizes with CS to further compromise the normal function of the intestinal barrier.

## Discussion

The significant roles of chronic stress and enteric pathogens in gastrointestinal diseases have garnered increasing attention (22, 32, 34). However, the synergistic mechanisms between these factors remain elusive. Clinical studies demonstrate that *L. monocytogenes* causes severe infections exclusively in immunocompromised patients and is enriched in tumor tissues of CRC patients (11–13), suggesting that this pathogen may exploit immunosuppressive environments to establish colonization and promote tumorigenesis through dysbiosis. Given the pathological progression from chronic inflammation to CRC, whether *L. monocytogenes* participates in the precancerous stage of CRC under specific host conditions remains systematically unexplored. This study provides experimental evidence in animal models that chronic stress facilitates *L. monocytogenes*-driven tumorigenesis by disrupting intestinal barrier integrity and recruiting MDSCs to the gut mucosa, thereby establishing a pro-tumorigenic immune microenvironment.

The expansion and recruitment of MDSCs are recognized as critical mechanisms underlying tumor immune evasion in multiple cancers (22, 24, 45). Under chronic stress stimulation, the spleen, the largest peripheral immune organ, exhibits significantly increased MDSCs proportions (45). We demonstrate that *L. monocytogenes* infection further amplifies chronic stress-induced generation of immature immune cells, indicating their synergistic modulation of systemic immunity.

The distribution of immune cells within hosts is influenced by multifactorial determinants. In tumor-bearing murine models, splenic MDSCs migrate to lesion sites via inflammatory signaling pathways, thereby promoting tumor progression (46). In this study, chronic stress and opportunistic pathogen infection recruited MDSCs to colonic tissues through elevated interleukin-6 (IL-6) levels. Notably, MDSCs proportion alterations in the spleen and colon displayed asynchronous dynamics. Splenic MDSCs did not traffic to the colon in the setting of *L. monocytogenes* infection alone. We propose that *L. monocytogenes* ruins microbial barrier function, while intact mucus layers, epithelial integrity, and TJs prevent microbiota and their metabolites from penetrating into the mucosa, thus failing to trigger MDSCs recruitment.

In TME, IL-6 secreted by stromal cells (e.g., T lymphocytes, macrophages, fibroblasts) binds MDSCs receptors to drive their recruitment (25, 38, 47, 48). Our data revealing synchronized trends between colonic IL-6 concentrations and MDSCs proportions, providing evidence supporting IL-6 as a pivotal mediator of MDSCs recruitment under chronic stress conditions.

MDSCs within TME exhibit potent immunosuppressive capabilities, primarily mediated through T-cell functional modulation (49). In colorectal tumor models, MDSCs suppress infiltration of CD8^+^ T cells and NK cells by upregulating arginase-1 (Arg1) activity (50, 51). Our prior work demonstrated that MDSCs stimulate Foxp3^+^ Treg proliferation via phosphorylation of the Stat3 pathway, thereby promoting colorectal carcinogenesis (23).

This study confirms that chronic stress combined with *L. monocytogenes* infection reduces CD8^+^ T-cell populations through enhanced MDSCs immunosuppressive function. Notably, *L. monocytogenes* infection alone did not induce significant local T-cell suppression in intestinal mucosal lesions. This may be attributed to the primary localization of *L. monocytogenes*-induced alterations within the intestinal lumen, where low-molecular-weight metabolites or inflammatory cytokines traverse the gut barrier to stimulate splenic MDSC expansion without activating mucosal recruitment mechanisms that require direct bacterial invasion or high-molecular-weight metabolite penetration.

Transcriptomic profiling revealed differential gene expression in MDSCs across experimental groups, with significant enrichment in tumor metabolism and immune-related signaling pathways. The cAMP pathway participates in regulating MDSCs immunosuppression across diverse malignancies (42). MDSCs in multiple cancer models overexpress cAMP signaling to activate STAT3-dependent T-cell inhibition. Furthermore, netrin-1 selectively secreted by colon cancer cells activates the cAMP/PKA cascade in MDSCs, amplifying their immunosuppressive activity (41). Notably, the significant enrichment of the “glutathione metabolism” and “mineral absorption” pathways suggests that chronic stress and *L. monocytogenes* infection disrupt immune homeostasis, potentially through modulation of MDSCs function. Specifically, the glutathione metabolism pathway is known to enhance the immunosuppressive capacity of MDSCs via glutamate signaling at metabotropic glutamate receptor 2/3 (52). Moreover, while minerals such as selenium and selenoprotein metabolites are established contributors to immune and inflammatory responses, the precise mechanisms underlying their beneficial effects remain incompletely defined (53).

We provide evidence that under sustained chronic stress, opportunistic pathogens can reshape immune responses to establish a pro-tumorigenic microenvironment conducive to intestinal adenoma development. Given MDSCs heterogeneity and interspecies variations, future investigations delineating human MDSCs subtype functions could advance targeted therapeutic strategies.

We demonstrate that the pro-tumorigenic effects of *L. monocytogenes* critically depend on chronic stress-induced disruption of barrier integrity. The outermost chemical barrier not only protects against microbial invasion but also modulates immune tolerance in the lamina propria (44). Our findings further indicate that chronic stress combined with *L. monocytogenes*, rather than pathogenic bacterium alone, primarily damages the mucus barrier.

Only after chronic stress compromises mucosal barrier integrity can *L. monocytogenes* and its pathogenic metabolites inflict subsequent damage (54). Moreover, although the microbial barrier formed by commensal flora adherent to the intestinal mucosa prevents colonization by exogenous pathogens under physiological conditions, its intimate contact with the gut wall may paradoxically increase invasion risks (55, 56). Published data confirm that gut dysbiosis disrupts host-microbiota interactions, promoting secretion of bacterial toxins and carcinogenic metabolites while reducing beneficial metabolites. This cascade impairs barrier function, induces immune dysregulation and cellular hyperproliferation, ultimately triggering CRC (57). Histopathological analyses corroborated that *L. monocytogenes* infection alone does not elicit epithelial dysplasia in the intestinal mucosa.

**Figure 5:**
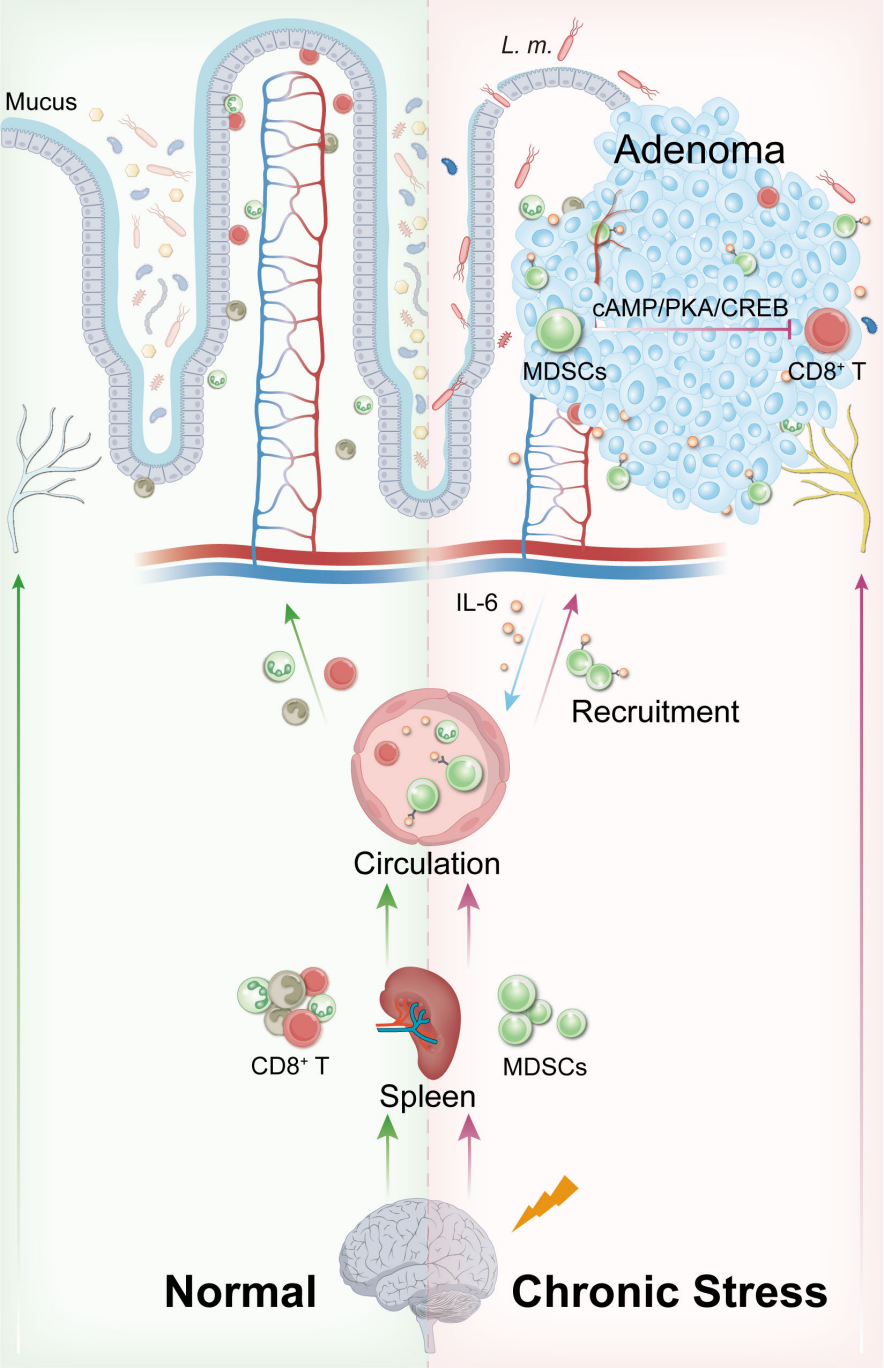
Schematic mechanism of chronic stress and *L. monocytogenes* synergistically promoting intestinal tumorigenesis via MDSCs.

Previous study by our group revealed intestinal dysbiosis in patients with recurrent colorectal polyps, even in histologically normal mucosa 10 cm away from the lesions. This suggests that gut microbiota dysregulation may be an early event in the formation of preneoplastic lesions (27). In the current study, we observed that chronic stress led to a reduction in beneficial bacteria (e.g., *Dubosiella*, *Prevotella* UCG-001, *Muribaculum*) and an increase in potentially harmful bacteria (e.g., *Helicobacter*, *L. monocytogenes*). The decline in the beneficial genus *Prevotella* is consistent with findings from prior clinical studies (27). Combined with elevated intestinal permeability, these results indicate that chronic stress, synergizing with *L. monocytogenes* infection, rather than opportunistic pathogen alone, significantly disrupts both the microbial and intestinal epithelial barrier, thereby exacerbating local immune responses (58–62) (**Figure 5**). This cascade of alterations creates a favorable microenvironment for the adhesion, colonization, and tumor-promoting effects of *L. monocytogenes*, further elucidating the mechanisms underlying the synergistic tumor-promoting effects of chronic stress and opportunistic pathogens.

## Data Availability

The original contributions presented in the study are publicly available. This data can be found here: https://dataview.ncbi.nlm.nih.gov/object/PRJNA1305642?reviewer=q5mmjlgsn27tgpve1slpej7ifu.

## Supplementary material

Supplementary Table 1 Antibody details.

Supplementary Table 2 Primer sequences.
