# Supplementary material for: Chronic stress synergizes with Listeria monocytogenes to promote intestinal adenomagenesis via myeloid-derived suppressor cells
Source: Front Immunol. 2025 Sep 3;16:1653548. doi: 10.3389/fimmu.2025.1653548 (PMC12440789; doi:10.3389/fimmu.2025.1653548)
Supplement: Supplementary Table 1 — Antibody details. [file Table1.docx]

Table S1：Antibody details

| Clone | Source | Identifier |
| --- | --- | --- |
| CD45-30-F11 | Biolegend | 103133 |
| CD11b-M1/70 | BD Biosciences | 557396 |
| GR1-RB6-8C5 | Biolegend | 108424 |
| CD3-145-2C11 | BD Biosciences | 553063 |
| CD4-RM4-5 | BD Biosciences | 553046 |
| CD8-53-6.7 | Biolegend | 100711 |
