# Supplementary material for: Chronic stress synergizes with Listeria monocytogenes to promote intestinal adenomagenesis via myeloid-derived suppressor cells
Source: Front Immunol. 2025 Sep 3;16:1653548. doi: 10.3389/fimmu.2025.1653548 (PMC12440789; doi:10.3389/fimmu.2025.1653548)
Supplement: Supplementary Table 2 — Primer sequences. [file Table2.docx]

Table S2：Primer sequences

| Gene | Sequence |
| --- | --- |
| Gadph | Forward（5’to 3’）：TCTGGAAAGCTGTGGCGTG  Reverse（5’to 3’）：CCAGTGAGCTTCCCGTTCAG |
| Occludin | Forward（5’to 3’）：TTGAAAGTCCACCTCCTTACAGA  Reverse（5’to 3’）：CCGGATAAAAAGAGTACGCTGG |
| Claudin | Forward（5’to 3’）：GGGGACAACATCGTGACCG  Reverse（5’to 3’）：AGGAGTCGAAGACTTTGCACT |
| ZO-1 | Forward（5’to 3’）：GCCGCTAAGAGCACAGCAA  Reverse（5’to 3’）：TCCCCACTCTGAAAATGAGGA |
| MUC2 | Forward（5’to 3’）：ATGCCCACCTCCTCAAAGAC  Reverse（5’to 3’）：GTAGTTTCCGTTGGAACAGTGAA |
